# The Efficient Disposal of Biomedical Waste Is Critical to Public Health: Insights from the Central Pollution Control Board Guidelines in India

**DOI:** 10.7759/cureus.47303

**Published:** 2023-10-19

**Authors:** Pratham P Gupta, Nandkishor J Bankar, Vaishnavi H Mishra, Shruti Sanghavi, Ankit K Badge

**Affiliations:** 1 Microbiology, Datta Meghe Medical College, Datta Meghe Institute of Higher Education and Research (Deemed to be University), Nagpur, IND; 2 Microbiology, Jawaharlal Nehru Medical College, Datta Meghe Institute of Higher Education and Research (Deemed to be University), Wardha, IND; 3 Ophthalmology, Datta Meghe Medical College, Datta Meghe Institute of Higher Education and Research (Deemed to be University), Nagpur, IND

**Keywords:** guidelines, biohazards, disposal, central pollution control board, biomedical waste management

## Abstract

Biomedical waste (BMW), encompassing hazardous medical materials, poses environmental and public health risks if not correctly managed. The Central Pollution Control Board (CPCB) in India is a statutory organization that oversees BMW disposal standards, aimed at mitigating these hazards. BMW mismanagement is a major problem and potentially poses threats to the environment as well as public health. During the coronavirus disease 2019 (COVID-19) pandemic, increased use of personal protective equipment (PPE) and other medical equipment was witnessed which led to a marked raised BMW generation. To ensure proper and optimized BMW management, CPCB established guidelines and rules to be followed by the medical facilities as well as the common BMW treatment facilities (CBWTFs). The challenges in implementing proper waste management practices were lack of awareness and inadequate infrastructure. Strategies for better BMW management were proposed, including color-coded bins, improved infrastructure, advanced technology, and awareness campaigns. Highlighting CPCB's vital role, this emphasizes healthcare facilities' proactive role in implementing and evolving regulations for sustainable BMW disposal, ensuring both public health and environmental well-being through compliance and responsible waste management partnerships.

## Introduction and background

Any waste created during human or animal diagnosis, treatment, or immunization is called biomedical waste (BMW). It contains syringes, needles, scalpels, blood, body parts, toxic chemicals, pharmaceuticals, medical devices, radioactive substances, and other potentially infectious objects [1]. The correct disposal of BMW is critical to public health; if not correctly disposed, it can lead to a substantial hazard to the environment and public health. In the Indian subcontinent, the Central Pollution Control Board (CPCB) has established standards to guarantee proper BMW disposal. It is a statutory organization under the Ministry of Environment, Forest and Climate Change (MoEFCC) of India, established in 1974 under the Water (Prevention and Control of Pollution) Act [2]. The healthcare delivery system, which was established to treat and protect people's health, is ironic because it has become a source of infection and a way of transmission of diseases [3]. The 3R concept, reduction, recycling, and reuse, is the foundation of BMW practice [4,5]. In India, CPCB is responsible for ensuring the proper and safe handling of BMW [6], which will be discussed in detail in this article. The article will also emphasize the negative consequences of improper handling and outline the steps that can be taken to ensure safe and appropriate handling. The study comprises reports from CPCB and data from various governmental and non-governmental organizations, revealing that the majority of states and unitary territories in India are facing concerns regarding the safe disposal of BMW.

## Review

Methods

To conduct a comprehensive literature search, we used the PubMed and Google Scholar advanced search strategy and websites to obtain articles from PubMed and Scopus using the following terms: (“Biomedical waste” OR “Bio medical waste” OR “Biomedical Waste Management” OR “Bio medical waste and public health” OR “Bio medical waste and environment” OR “bio medical waste and Indian laws” OR “bio medical waste and disposal” OR “biomedical waste management” OR “biomedical waste and handling”).

Articles Screened

**Figure 1:**
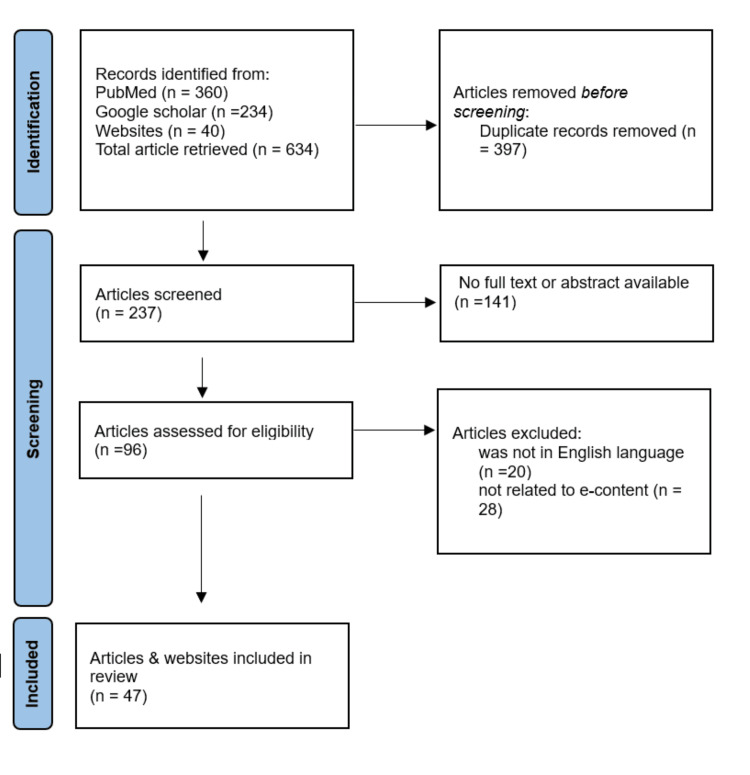
PRISMA flowchart PRISMA: Preferred Reporting Items for Systematic Reviews and Meta-Analysis; n: number of studies

We focused on works relevant to our review's key topics, including review articles and randomized clinical trials. We excluded those unrelated to BMW. After screening, we retained 47 publications from an initial pool of 634, eliminating 397 duplicates and some studies lacking full texts or related content (Figure 1).

BMW generation scenario in India before and after the coronavirus disease 2019 (COVID-19) pandemic

Karnataka, Maharashtra, Tamil Nadu, Uttar Pradesh, and Kerala were the top five states in India in terms of total BMW generation prior to the COVID-19 pandemic. The state of Karnataka produced the most BMW (77.5 tons/day), whereas the least generation was by Arunachal Pradesh (0.4 tons/day) [7]. Among all the union territories of India, Delhi generated the highest amount of BMW (28.8 tons/day), while Lakshadweep produced the least amount (0.1 tons/day). Annually, India generated BMW at a rate ranging from 0.5 to 2.0 kg/bed per day [8].

Since the COVID-19 pandemic, BMW has been significantly altered due to the increased use of personal protective equipment (PPE) and medical technologies [9]. Tamil Nadu, Gujarat, Maharashtra, Delhi, Haryana, West Bengal, Kerala, Madhya Pradesh, Uttar Pradesh, and Punjab were the major states and union territories that contributed to BMW generation during the first wave (September 2020), making up nearly 80% of the total BMW [10]. The average BMW generation was 183 tons/day during September 2020 and 203 tons/day during the second wave (May 2021) [11]. As of August 2022, Kerala (1.15 tons/day), Delhi (0.72 tons/day), Maharashtra (0.44 tons/day), Tamil Nadu (0.41 tons/day), Haryana (0.37 tons/day), and Gujarat (0.26 tons/day) were the major contributors of BMW [11].

CPCB guidelines

The CPCB in India established guidelines for the proper disposal of BMW. These recommendations are meant to protect the environment and stop the transmission of infectious diseases. BMW management regulations offer a structure for handling BMW, from generation to disposal. The amendments made in the BMW management rules are mentioned in Table 1.

**Table 1:** Changes in biomedical waste management rules CBWTF: common biomedical waste treatment facility; GPS: Global Positioning System References: [12-14]

Year	Regulation	Key provisions
1989	Biomedical Waste (Management and Handling) Rules, 1998	Defined categories of biomedical waste. Mandated segregation, collection, and disposal of biomedical waste. Required the establishment of a disposal facility. Set standards for the treatment and disposal of biomedical waste. Prescribed labeling and packaging requirements.
2000	Amendment to 1998 Rules	Included specific requirements for the disposal of laboratory waste and microbiological waste. Introduced color-coding for waste categories. Emphasized the need for training and awareness programs.
2003	Amendment to 1998 Rules	Extended the coverage of the rules to include blood banks, veterinary hospitals, and animal research institutions. Mandated using autoclave for treating infectious waste. Introduced the provision for a CBWTF.
2011	Biomedical Waste (Management and Handling) Rules, 2011	Replaced the 1998 Rules and consolidated amendments. Added more categories of waste and specific waste management provisions. Enhanced regulatory requirements for CBWTFs. Emphasized waste minimization and training. Introduced provisions for annual reporting and records maintenance.
2016	Amendment to 2011 Rules	Revised the categorization of biomedical waste into four color-coded categories. Included the category of "Pharmaceutical Waste." Set stricter emission standards for incinerators at CBWTFs. Stipulated more stringent standards for the treatment of biomedical waste. Mandated the use of barcoding and GPS for tracking waste. Enhanced reporting and monitoring requirements.

Changes introduced due to COVID-19

Due to the dramatic growth of the BMW generation, CPCB has taken additional measures to improve the management of BMW throughout the COVID-19 pandemic [15]. In addition to the existing practice under the 2016 BMW Management guidelines, stakeholders must follow these guidelines [16]. These include the role of stakeholders in troubleshooting problems of laboratory waste, PPE waste, home care waste, solid waste, liquid waste, and medical and BMW workers [17]. In July 2020, the fourth revision of the guidelines was published, which included updates and modifications for separating general solid waste and BMW from quarantine centers, home care facilities, and hospitals treating COVID-19 patients, as well as recommendations for the disposal of PPE [16].

Importance of proper BMW disposal

BMW contains anatomical waste, sharps, laboratory waste, and other waste, which can be fatal if not carefully separated. Furthermore, improper separation of polluted plastics, which is a cytotoxic and recyclable material, can damage our ecosystem [18]. BMW is naturally dangerous because it consists of possible viruses or other microbial particles and can exist in human samples, blood bags, needles, cotton swabs, clothing, bedding, etc. Thus, inadequate management of BMW is a community health problem [6]. Proper management is designed to reduce environmental pollution because BMW can pollute air, water, and land if not properly managed [6]. Improper disposal of BMW can have severe consequences. For example, if such waste is dumped in the open, it can attract animals, birds, and insects that can spread the waste further. Therefore, efficient BMW disposal can reduce these concerns and preserve human health and the environment.

Risk of improper BMW handling

The main risk groups are healthcare workers, excavators, and the public. Microbial-related infections caused by BMW and sample exposure include both systemic and local medical conditions [19-21]. Mercury, disinfectants, and pesticides have an impact on multiple systems. Improper handling of needles leads to needle wounds and infection of pathogens that carry blood such as hepatitis B virus (HBV), human immunodeficiency virus (HIV), and hepatitis C virus (HCV) [21-23]. Ensuring the safe and environmentally sound management of healthcare wastes can prevent adverse health and environmental impacts including potential infectious risks from multidrug-resistant microorganisms, which may lead to the spread of infections like multidrug-resistant tuberculosis (MDR-TB), thus protecting the health of the health workers and the general public [21]. The various risk factors and their impact on humans, animals, and the environment are mentioned in Table 2.

**Table 2:** Potential risk of biomedical waste due to improper handling HIV: human immunodeficiency virus; DNA: deoxyribonucleic acid References: [19-23]

Risk factor	Impact on humans	Impact on animals	Impact on environment
Infectious agents	Risk of infections (e.g., HIV, hepatitis)	Spread of diseases	Contamination of soil and water
Chemical hazards	Exposure to toxic substances	Poisoning or illness	Soil and water pollution
Radioactive materials	Radiation exposure	Radiation sickness	Contamination of soil and water
Sharps	Needlestick injuries	Infection and injury	Environmental hazard
Pharmaceuticals	Drug contamination	Unknown effects	Water pollution, residue
Heavy metals	Poisoning (e.g., lead, mercury)	Harmful effects	Soil and water contamination
Pathological waste	Disease transmission	Scavengers may get infected	Contamination of soil and water
Genotoxic waste	DNA damage	Genetic mutations	Potential carcinogens
Microplastics	Ingestion and health issues	Ingestion and health issues	Marine and terrestrial pollution
Chemical runoff	Water pollution	Water contamination	Aquatic ecosystem damage
Air-borne particles	Respiratory issues	Respiratory issues	Air pollution
Radioactive waste	Potential for improper disposal	Contamination of ecosystems	Soil contamination

Effects and consequences of BMW on public health

If BMW is mishandled, it may be to blame for the production of numerous large-scale vectors that accelerate the spread of vector-borne diseases. Additionally, it contaminates land and water and spreads pandemics and diseases, such as acquired immunodeficiency syndrome (AIDS) and COVID-19, through contaminated syringes and needles. Improper disposal of BMW can also lead to the spread of antibiotic-resistant bacteria. Such bacteria can cause serious infections that are difficult to treat [4]. BMW management issues and garbage mixing lead to soil, water, and air pollution. Therefore, it contributes to infectious diseases and poor health. Today, the relationship between BMW generation and therapy is inverted, that is, BMW generation is very high and its treatment rate is relatively very low. Such scenarios indicate that our country will soon sink into its waste [23].

Challenges in implementing proper BMW disposal practices

Lack of Awareness and Training

One of the primary challenges is the lack of awareness and training among healthcare workers and waste handlers on proper waste management practices. Insufficient knowledge about waste segregation, handling, and disposal can lead to inappropriate practices and potential risks to public health and the environment [24].

Inadequate Infrastructure and Resources

Many healthcare facilities, particularly in resource-constrained settings, face challenges in establishing and maintaining the appropriate infrastructure and resources for the handling of BMW. The lack of dedicated waste disposal units, insufficient waste collection systems, and limited treatment facilities can hinder proper waste management [25].

Compliance and Enforcement Issues

Ensuring compliance with waste management regulations can be challenging, especially in areas where enforcement mechanisms are weak. Inadequate monitoring, inspection, and penalties for noncompliance can undermine the effectiveness of waste management practices [26].

Informal Sector Involvement

The informal sector's engagement in collection and disposal raises issues. Informal waste pickers often handle BMW without proper protective measures, raising the danger of exposure to hazardous materials and the spread of infectious diseases [27]. CPCB should also consider publishing the guidelines in the local dialect according to the region, apart from Hindi and English.

Strategies and solutions for better BMW management

An illustration of proper BMW was recorded in 1998, and the British Ministry of Health [28] drafted a document stating that "health authorities, health councils, and National Health Service (NHS) trusts are responsible for establishing their own rules for mitigating the spread of blood-borne viruses (BBV) in the healthcare environment" [29]. The lack of waste management infrastructure can be considered a major challenge faced by developing countries like India [9]. Robust and sustainable methods are required to address the challenge of immense garbage generated by COVID-19. To combat the pandemic, better technologies and sustainability are needed. Some plans and efforts necessitate immediate attention as well as action, while others necessitate significant long-term commitment [30]. Single-use plastics (SUP) and BMW must be safely disposed of to prevent the spread and limit environmental damage. To ensure the proper collection and disposal of bins, color-coded bins should be used [3,29].

In the context of the pandemic, BMW management stakeholders should build more advanced and automated systems (based on the Internet of Things), reducing the number of workers involved and taking into account public health and safety [31]. It is necessary to mention and adopt methods for the separation, collection, storage, transportation, and disposal of waste produced with regard to the BMW management guidelines [32,33]. Investment in healthcare facilities and the recruitment of trained health workers are critical to increasing treatment capacity [34,35]. Different methods of disinfection such as hydrogen peroxide steam, washing, ultraviolet (UV) disinfection lamps, humidifiers, gamma radiation, alcohol solutions of 75%, and ethylene oxides can be considered [36-41]. Sustainable and recyclable goods should be promoted [42]. Modern techniques like high-temperature hydrothermal carbonization and autoclave pressure technology can be used to carbonize BMW [43,44]. A public awareness program and a media campaign must be launched to raise awareness of the environmental impact of accidental discharges and poor governance of plastic waste (PW) and BMW. Furthermore, the environmental implications of BMW and PW should be incorporated into the school curricula to create awareness among generations to come [9].

In the Indian setting, CPCB, the government body responsible for the formulation of the guidelines for the proper handling of BMW, has advised the following strategies for better BMW management: Under the 2016 BMW Disposal Regulation, color-coded boxes or containers are placed in the classroom to properly organize BMW [45]. Before the disposal and immediate disposal of the waste, it is necessary to provide a well-ventilated intermediate storage area for BMW separately [45]. In particular, waste from COVID-19 isolation rooms should be collected in two layers of bags to avoid bag leakage [46]. Depending on the type of waste received by the CBWTF, it must be disposed of appropriately by the type of waste [47].

Responsibilities of healthcare facilities in BMW management

Healthcare facilities should have a designated team for the proper management and disposal of BMW, and their duties are mentioned in Table 3.

**Table 3:** Healthcare professionals' duties towards the disposal of biomedical waste

Healthcare professionals	Duties
Hospital project manager	Assign the local waste manager to lead the working group in charge of developing the waste management plan, who will oversee and coordinate the waste management plan daily.
Hospital administrator	Ensure that consumable supplies are readily available. Make purchase recommendations to reduce/replace specific items. Keep an eye on safety precautions.
Head nurse	Educate carers about trash management. Monitor safety precautions and procedures in various wards.
Chief pharmacist	Maintain pharmaceutical supply records and reduce outdated stock. Take care of products containing mercury.
Head of laboratory	Maintain chemical records and reduce chemical waste. Minimize chemical waste.

Healthcare facilities bear the responsibility of effective BMW management to ensure public health and environmental safety. This involves the segregation, proper collection, transportation, treatment, and disposal of BMW. Adhering to regulations, using appropriate containers, and implementing training programs for staff are crucial. By minimizing health risks to workers, patients, and communities, healthcare facilities play a vital role in maintaining a hygienic and ecologically sound environment. 

## Conclusions

Recognizing that proper BMW disposal is pivotal for public health and environmental preservation, it is essential to underscore the role of CPCB regulations in India. Effective BMW disposal during the COVID-19 pandemic is essential for infection prevention as well as for the avoidance of negative effects on the environment and public health. Moreover, healthcare facilities should take a proactive stance in adopting and evolving these regulations to ensure a sustainable future with minimized environmental and public health risks. Continued compliance with evolving best practices and government regulations, in conjunction with the services of BMW disposal companies, paves the way for a sustainable and environmentally conscious future.
